# Combinatorial Epigenetic and Immunotherapy in Breast Cancer Management: A Literature Review

**DOI:** 10.3390/epigenomes4040027

**Published:** 2020-12-04

**Authors:** Yu-Ting Lee, Yu-Ming Chuang, Michael W. Y. Chan

**Affiliations:** 1 Department of Biomedical Sciences, National Chung Cheng University, Min-Hsiung, Chia-Yi 621, Taiwan; ccu403250029@gmail.com; 2 Epigenomics and Human Disease Research Center, National Chung Cheng University, Min-Hsiung, Chia-Yi 621, Taiwan; 3 Center for Innovative Research on Aging Society (CIRAS), National Chung Cheng University, Min-Hsiung, Chia-Yi 621, Taiwan; 4 Division of Hematology and Oncology, Department of Medicine, Ditmanson Medical Foundation Chiayi Christian Hospital, Chia-Yi 621, Taiwan

**Keywords:** immune checkpoint inhibitors, epi-drug, combination therapy, breast cancer

## Abstract

Breast cancer is one of the leading causes of death among cancer patients worldwide. To date, there are several drugs that have been developed for breast cancer therapy. In the 21st century, immunotherapy is considered a pioneering method for improving the management of malignancies; however, breast cancer is an exception. According to the immunoediting model, many immunosuppressive cells contribute to immunological quiescence. Therefore, there is an urgent need to enhance the therapeutic efficacy of breast cancer treatments. In the last few years, numerous combinatorial therapies involving immune checkpoint blockade have been demonstrated that effectively improve clinical outcomes in breast cancer and combining these with methods of targeting epigenetic regulators is also an innovative strategy. Nevertheless, few studies have discussed the benefits of epi-drugs in non-cancerous cells. In this review, we give a brief overview of ongoing clinical trials involving combinatorial immunotherapy with epi-drugs in breast cancer and discuss the role of epi-drugs in the tumor microenvironment, including the results of recent research.

## 1. Introduction

Breast cancer, a malignancy of mammalian cells, is the most commonly diagnosed cancer in females and one of the leading causes of cancer death worldwide [1]. Major molecular subtypes of breast cancer include luminal A, luminal B, HER2 enriched, and basal-like (or triple-negative breast cancer; TNBC) [2,3]. In clinical practice, some immunohistochemical (IHC) markers, including estrogen receptor (ER), progesterone receptor (PR), human epidermal growth factor receptor 2 (HER2), and Ki-67, can function as surrogate markers capable of identifying molecular subtypes [4,5].

Management of breast cancer is provided by multidisciplinary teams. Early-stage breast cancer is treated by surgical resection followed by neoadjuvant/adjuvant chemotherapy, radiotherapy, endocrine therapy, or anti-HER2 therapy. Systemic therapy for metastatic breast cancer includes endocrine therapy, target therapy and cytotoxic chemotherapy. The treatment option for metastatic breast cancer is on the basis of the cancer subtype (Table 1) [5,6]. Endocrine therapy, involving selective estrogen receptor modulators, such as tamoxifen and fulvestrant, and aromatase inhibitors, such as anastrozole, letrozole, and exemestane, is used to treat hormone receptor (HR)-positive breast cancer. Although endocrine therapy can extend the median progression-free survival (PFS) to up to 24 months, resistance to the treatment may occur [7]. One mechanism of endocrine resistance is the upregulation of the PI3K/AKT/mTOR pathway [8]. A phase III study (BOLERO-2) demonstrated that the mTOR inhibitor everolimus, combined with exemestane, can overcome endocrine resistance [9]. Alpelisib is a PI3K inhibitor used as a targeted therapeutic drug. Combined therapy of alpelisib and fulvestrant improved PFS among patients with PIK3CA-mutated, HR-positive, HER2-negative breast cancer who had received endocrine therapy previously [10].

Cyclin D1 is one of the main regulators of the cell cycle. Cyclin D1 amplification is a common oncogenic event in breast cancer, especially in luminal tumors [11]. Overexpression of cyclin D1 in breast cancer cells is also another mechanism of endocrine resistance [12]. Recently, a CDK4/6 inhibitor combined with fulvestrant or aromatase inhibitor was shown to markedly increase PFS and overall survival (OS), and will become an upfront therapy for hormone receptor (HR)-positive breast cancer [13,14,15,16]. For HER2-positive breast cancer, multiple anti-HER2 agents have been developed. These drugs include monoclonal antibodies (trastuzumab and pertuzumab), small molecule tyrosine kinase inhibitors (lapatinib and neratinib), and the antibody-drug conjugate (ADC) ado-trastuzumab emtansine (T-DM1) [6]. Finally, TNBC is usually treated by cytotoxic chemotherapy. Patients with germline BRCA1/2 mutations or homologous recombination deficiency may benefit from Poly-ADP ribose polymerase (PARP) inhibitors [17,18]. In the 21st century, immunotherapy was considered one of the groundbreaking methods for cancer treatment.

Compared to other subtypes, TNBC is more sensitive to checkpoint inhibitors, but the clinic benefit is controversial [19]. In 2018, the IMpassion130 study demonstrated that atezolizumab, an anti-PD-L1 monoclonal ab, plus chemotherapy, improves survival among patients with TNBC whose tumors express PD-L1 [20]. IMpassion131 was similar to Impassion 131 and evaluated atezolizumab in combination with alternative chemotherapy regimen. However, the IMpassion131 study showed that the addition of atezolizumab did not improve PFS and OS. On the contrary, a trend toward better survival in the placebo group was observed (NCT03125902). The role of immunotherapy treating breast cancer is under investigation.

Although the first antibody-based immunotherapy, anti-HER2, was approved in 1998, resistance and relapse remain major barriers in breast cancer treatments. Novel approaches based on combinatorial immunotherapy may act as therapeutic strategies to overcome these impediments. In this review, we summarize recent findings in the combinatorial immuno- and epigenetic therapy in the treatment of breast cancer.

## 2. Epigenetics and Epi-Drugs

Epigenetics was first described in 1942 by C. H. Waddington [23]. Different from genetics, epigenetics involves the control of heritable phenotypes by post-modifications rather than DNA sequence alteration. Epigenetic modifications also reflect the communication between environment and host factors, as most epigenetic processes require distinct metabolites as substrates, such as acetyl-CoA and α-ketoglutarate, which are generated from the environment [24]. Epigenetic modifications can be briefly classified by the application of the central dogma of molecular biology to DNA methylation, histone modification, and non-coding RNA regulation. Over the past 30 years, an aberrant epigenetic landscape has been defined as a hallmark of cancer [25]. Understanding the epigenetic mechanism is necessary for the development of epi-drugs for targeting epigenetic regulators as a promising strategy for anticancer treatment [26].

DNA methylation is a covalent chemical modification, a common form of epigenetic regulation and response to environmental stress. It involves the modification of the fifth carbon of cytosine by the addition of a methyl group and plays an important role in regulating gene transcription. DNA methylation is also involved in important processes in development, aging, and health, such as X-chromosome inactivation, C to T transition mutation, and silencing of repeated sequences [27]. DNA methylation and demethylation are regulated by DNA methyltransferases (DNMTs) and ten-eleven translocation methylcytosine dioxygenases, respectively [28].

Histone modifications, on the other hand, result in conformation and charge changes of the chromatin, which regulate gene transcription or chromatin interaction [29]. The regulators associated with histone modifications are classified as writer, reader, and eraser, which function via chemical group addition, recognition, and elimination on the histone tail, respectively [30]. Histone lysine methylation and acetylation are the major modifications, which have been studied widely in cancers. Mono-methylation and tri-methylation at lysine 4 of histone H3, forming H3K4me1 and H3K4me3, are active enhancer and promoter marks, respectively. On the other hand, tri-methylation of lysine 27 of histone H3, catalyzed by EZH2, one of the members in polycomb repressive complex 2 (PRC2), is a repressive mark [31]. Acetylation of lysine on histone, which is catalyzed by histone acetyltransferase and histone deacetylase (HDAC), activates transcription and can be recognized by bromodomain-containing proteins [32,33].

Generally, several inhibitors targeting epigenetic writers, readers, and erasers have been developed and clinical trials of those epi-drugs are ongoing. For example, vorinostat, an HDAC inhibitor, is used to treat relapse/refractory cutaneous manifestations of cutaneous T-cell lymphoma [34], and DNMT inhibitors, including decitabine and azacitidine, are available for myelodysplastic syndrome and some acute myeloid leukemia [35,36].

The antitumor effects of epi-drugs encompass cytotoxic effects, apoptosis, growth arrest, differentiation, inhibition of angiogenesis, and immunogenicity [37,38]. Epigenetic therapies may reactivate the expression of genes that have undergone epigenetic silencing, thereby reprogramming the cancer cells [39]. Interestingly, either ER or PR was epigenetically silenced by DNMT and HDAC in breast cancer, and could be restored by epi-drugs [40,41]. However, in contrast to hematologic malignancy, the efficacy of monotherapy with epi-drugs is unsatisfactory in solid tumors. Epi-drugs might have the potential for synergistic or additive effects to augment the efficacy of antitumor drugs or to overcome drug resistance [37]. In this review, we focus on the combination of epi-drugs and immunotherapy in the treatment of human cancer.

## 3. Rationale for Epi-Drugs Combined with Immunotherapy

Immune checkpoint blockade (ICB) is one of the major forms of cancer immunotherapy. The programmed cell death 1 (PD-1) receptor on the surface of immune cells interacts with its ligand, programmed cell death ligand 1 (PD-L1), which can be produced by tumor cells or immunosuppressive cells, and suppresses the cytotoxic function of T-cells. Antibodies that bind to either PD-1 or PD-L1 can reactivate the immune response of preexisting T-cells [42].

However, the response rate of anti-PD-1/anti-PD-L1 inhibition in breast cancer is around 5–10%, and thereafter combination therapy is more practicable for breast cancer [19,20,43]. For example, pembrolizumab plus neoadjuvant chemotherapy increased the proportion of pathological complete response in patients with early TNBC [44]. These results suggest that combination therapies with ICB are very promising. Additionally, the tumor microenvironment has been shown to determine the outcome of cancer treatment. Therefore, a shift in therapeutic strategies from targeting tumor cells to the tumor microenvironment is required [45,46]. On the whole, it is believed that breast cancer is immunologically quiescent, due to a low infiltration of effector cell, tumor mutation burden (TMB), and response rates to ICB [47]. The main mechanisms of tumor evasion include alteration in antigen presenting cells (APCs), dysfunction of effector cells, and changes in tumor cells [48]. Recently, single-cell transcriptomic analysis revealed the heterogeneity of breast cancer in detail. In breast cancer, immunosuppressive macrophages, regulatory T-cells and exhausted T-cells are enriched [49,50]. Previous studies have found that epigenetic changes also control the homeostasis of immune cells and contribute to immunosuppressive phenotypes [51,52]. As human cancers are heterogeneous, the mechanisms by which epi-drugs contribute to non-cancerous cells for cancer treatments are still under investigation. Below, we review ongoing and completed clinical trials involving epi-drugs combined with other therapies, including immunotherapy, in breast cancer (Table 2), and discuss the potential mechanism by which epi-drugs target the microenvironment of breast cancer.

### 3.1. Targeting DNA Methylation

Azacitidine and decitabine are cytidine analogues that inhibit DNA synthesis at high doses and inhibit DNA methyltransferase at low doses, leading to hypomethylation of DNA [64]. A phase I study of the inhibition of DNA methylation did not show benefits for breast cancer [53,54,55]. A phase II trial was conducted to examine a combination of epigenetic therapy, azacitidine, and a histone deacetylase inhibitor (entinostat) in women with advanced breast cancer. There was one partial response among 27 women with hormone-resistant disease (ORR: 4%), and none in 13 women with TNBC [65].

Inhibiting DNA methylation triggers endogenous retrovirus (ERV) expression, which is usually silenced by DNA hypermethylation. The released dsDNAs are targeted as tumor-associated antigens [66] or are sensed by the cGAS DNA-sensing pathway and further induce type I interferon to engage T-cell infiltration [67]. One study indicated that ERV expression shows a highly positive correlation with immune cytolytic activity in breast cancer [68]. Recently, Panda et al. found that the expression of ERVs was related to the ICB response in clear cell renal cell carcinoma [69], which suggests that a combination of DNMTi with ICB may also improve clinical outcomes in breast cancer. In addition to ERVs, de novo exhaustion-related DNA methylation was a progressive program in effector T-cells during ICB, and inhibition of DNA methylation reversed the progression and improved T-cell rejuvenation [70].

Recently, combinations of DNA methylation inhibitors and immunotherapy have been under investigation. A phase II study (NCT02811497) will assess the antitumor activity of azacitidine in combination with durvalumab for participants with metastatic ER-positive/HER2-negative breast cancer. Another phase II (NCT02957968) trial is recruiting and will examine short-term neoadjuvant therapy with pembrolizumab plus decitabine for patients with locally advanced HER2-negative breast cancer.

### 3.2. Targeting Histone Deacetylation

Clinical data for breast cancer treatment using HDAC inhibitor monotherapy are rare [71]. Some phase II trials showed that combining HDAC inhibitors with chemotherapy is a safe strategy. The clinical benefit is observed among heavily pretreated patients, suggesting that addition of an HDAC inhibitor may overcome the resistance to chemotherapy [56,72,73]. Furthermore, breast cancer therapy combined with HDACi also reversed hormone therapy resistance [57,58]. In terms of immunotherapy, Munster et al. reported using tamoxifen in combination with vorinostat and pembrolizumab in the treatment of hormone therapy-resistant breast cancer. A clinical benefit was seen in 5/28 (18%) patients (NCT02395627) [74]. Unfortunately, clinical data for the combination of HDAC inhibitors with immunotherapy are limited. Further clinical trials are ongoing (Table 2).

The role of HDACs has been well investigated in T-cell homeostasis. A previous study demonstrated that loss of HDAC1 and HDAC2 promotes the effector T-cell program by upregulating RUNX3 [75]. Moreover, HDAC11-deficient CD4^+^ T-cells showed increased expression of Eomes and Tbet, which induced effector phenotypes and inflammatory cytokine secretion [76]. Along with effector T-cells, HDACs also activate the function of regulatory T-cells. The major regulator, FOXP3, interacts with HDAC3 to suppress IL2. Loss of HDAC3 in regulatory T-cells restores IL2 expression and increases the levels of other inflammatory cytokines [77]. Nevertheless, some studies showed conflicting results of other HDACs in T-cell hemostasis [78,79], suggesting that targeting specific HDAC may be necessary.

### 3.3. Targeting Histone Demethylation

LSD1, also known as KDMA1, is a histone demethylase controlling H3K4 and H3K9 demethylation, which is also a novel therapeutic target in cancer. Numerous inhibitors under ongoing clinical trials have been reported to-date in small cell lung cancer and acute myeloid leukemia [80]. In breast cancer, clinical trials are being performed to investigate the potential of phenelzine sulfate, an LSD1 inhibitor, combined with nanoparticle albumin-bound paclitaxel, Abraxane, for metastatic or advanced tumor (NCT03505528). Unfortunately, clinical trials involving combinatory immunotherapy with LSD1 inhibitor, INCB059872, have only been undertaken for other cancers (NCT02712905).

LSD1 has been reported as a key regulator for stemness and chemo-resistance in breast cancer [81]. Furthermore, targeting LSD1, which causes increased H3K4 methylation, could also cooperate with ICB by ERV-mediated T-cell trafficking in breast cancer [82,83]. Additionally, inhibition of LSD1 can epigenetically reprogram tumor-associated macrophages into M1-like macrophages by increased methylation on H3K4 and H3K9, and disrupt the LSD1–CoREST complex in TNBC [84]. In addition to KDM1A, Wu et al. reported that stimulator of interferon genes (STING), which related to intratumoral CD8^+^ T-cells, are epigenetically silenced by KDM5B and KDM5C, leading to immune quiescence. These findings suggest that targeting histone demethylation plus ICB may be a viable new strategy for treatment of breast cancer.

### 3.4. Targeting Histone Methylation

EZH2 is a well-known oncogenic histone methyltransferase. Overexpression of EZH2 has been frequently observed in breast cancer and is associated with breast cancer aggressiveness and the sensitivity to chemotherapy [85,86]. Emerging trials of EZH2 inhibitors are ongoing but mainly limited to hematologic malignancy and soft tissue sarcoma [87].

The inhibition of EZH2 is a double-edged sword for immunotherapy. Peng et al. found that EZH2-mediated H3K27me3 and DNMT1-mediated DNA methylation repress the ability of ovarian tumor to present Th1-type chemokines CXCL9 and CXCL10. Treatment with EZH2i or DNMTi reactivates these chemokines and allows effector T-cell tumor trafficking in the tumor microenvironment [88]. In contrast, Huang et al. reported that suppressing EZH2 activity promotes hematopoietic progenitor cell differentiation to myeloid-derived suppressor cells (MDSCs), and increases MDSCs in the tumor microenvironment, thus suppressing antitumor immunity [89]. Clinical trials for combination of EZH2 inhibition with immunotherapy have also been initiated in urothelial carcinoma [90], but for breast cancer have not yet been initiated.

### 3.5. Targeting Bromodomain in Breast Cancer

Bromodomain and extraterminal (BET) inhibition, which disrupts the linkage between enhancer and promoter for transcriptional repression, is a novel therapy for treating breast cancer. The pharmacokinetics and pharmacodynamics of several BET inhibitors, including birabresib, molibresib and mivebresib, have been established [62,63,91]. However, the toxicities of these BET inhibitors are notable. In a phase I study assessing the safety and pharmacokinetics of BET inhibitor mivebresib in patients with non-specific relapsed or refractory solid tumors, 62/84 (74%) patients had a grade III/IV treatment-emergent adverse event (AE), and all grades of AEs were reported across 81/84 (96%) patients [63]. Another phase I/II dose-escalation study for BET inhibitor molibresib on selected solid tumors, including TNBC, showed 54/65 (83%) patients had a treatment-related AE [62]. Therefore, the drug-related toxicities may limit the usage of combination. Further safety profile and clinical efficacy tests have been initiated in TNBC (NCT03901469) and other tumors (NCT03925428, NCT04116359).

In the immune system, treatment of the BET inhibitor can differentiate effector T-cells into the effector memory phenotype by suppressing BATF [92]. Additionally, Adeegbe et al. reported that BET inhibition attenuates the function of regulatory T cells, and cooperates with PD-1 blockade to facilitate an antitumor response in lung cancer [93,94]. Furthermore, BET inhibition will suppress both PD-1 in effector T cells and PD-L1 in breast cancer cells to overcome tumor-mediated T cell exhaustion in TNBC [95]. These findings suggest the probable effectiveness of combinatory immunotherapy with bromodomain inhibition, especially in targeting T cells. Interestingly, a mathematical model likewise supports the benefit of the BET inhibitor combined with anti-CTLA4 which sustains cytotoxic T cell in tumors [96].

## 4. Conclusions

**Figure 1:**
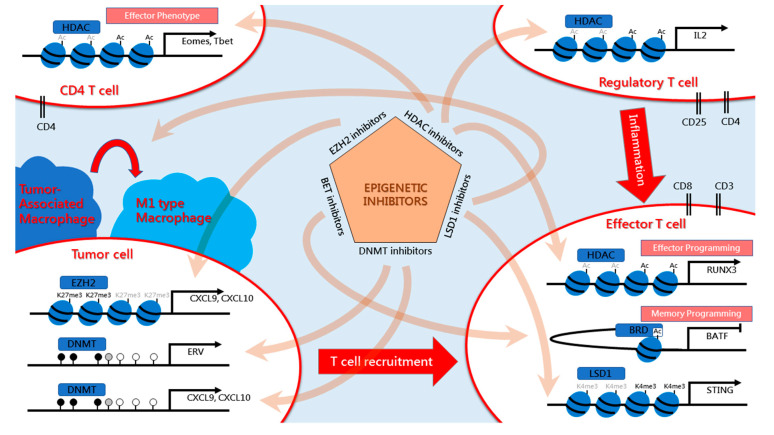
Summary of epigenetic inhibitors in tumor microenvironment for overcoming immune evasion.

A growing body of evidence suggests the probable effectiveness of combinations of epi-drugs with immunotherapy in breast cancer treatment. To overcome the immune evasion of cancer, targeting both tumor cells and the microenvironment is crucial. Therefore, the mechanism of homeostasis of immune cells and the communication between tumor and microenvironment in breast cancer require further investigation. In addition, the heterogeneities of breast cancer are distinct between cancer subtypes, suggesting that it is necessary to investigate the distinct epigenetic dysregulation of non-cancerous cells in the breast tumor microenvironment. In this review, we generally discussed combinatory immunotherapy with epi-drugs, including those involved in DNA methylation, histone modification, and bromodomain inhibition, in several ongoing clinical trials and basic medical research (Figure 1). Additionally, non-coding RNA also can be considered as a therapeutic target. Recently, clinical trials in mesothelioma and lymphoma have begun for several miRNA-based drugs, such as the mir-16 mimic, MesomiR-1, and the mir-155 inhibitor, MRG-106, but the clinical outcomes associated with these two drugs require further investigation in breast cancer. Finally, further investigation into whether combined differential epi-drugs plus ICB for specific targeting of each kind of immunosuppressive cells can be of greater benefit in breast cancer, and how to precisely target specific cells in the tumor microenvironment to prevent conflicting effects, is required. The development of precisely targeted drug delivery systems may improve these novel therapeutic approaches for breast cancer therapy.

## Figures and Tables

**Table 1:** Summary of systemic treatment for metastatic breast cancer.

Subtype Incidence [21,22]	Pathological Definition ^1^	Systemic Therapies
HR(+)/HER2(−) 60–70%	ER or PR >1% of cells staining	Endocrine therapy-based regimens
		First line of therapy AI + CDK4/6 inhibitor Endocrine therapy (AI, SERD, SERM) Subsequent-Line Therapy AI + CDK4/6 inhibitor mTOR inhibitor + endocrine therapy: Everolimus + endocrine therapy (exemestane, fulvestrant, or tamoxifen) PI3K inhibitor + endocrine therapy ^2^: Alpelisib + fulvestrant Endocrine therapy (AI, SERD, SERM) Chemotherapy PARP inhibitors ^3^
HER2 (+) with HR (+) or HR (−) 15%	HER2 staining: strongly positive (3+) or HER2 gene amplification by in situ hybridization assay	First line of therapy Dual HER2-blockade + Chemotherapy: Pertuzumab + trastuzumab + Taxanes Subsequent-Line Therapy Ado-trastuzumab emtansine Anti-HER2 + chemotherapy
Triple-Negative 15%	Negative ER, PR, and HER2	Chemotherapy Immune checkpoint inhibitor + chemotherapy Atezolizumab + albumin-bound paclitaxel PARP inhibitors ^3^

^1^ ASCO/CAP guidelines (PMID: 29,846,122 and 31928404): ^2^ for tumors with *PIK3CA* mutation tumors, ^3^ for germline *BRCA1/2* mutations. ER: estrogen receptor; PR: progesterone receptor; HER2: human epidermal growth factor receptor 2; AI: aromatase inhibitor; SERM: selective estrogen receptors modulator; SERD: selective ER down-regulator.

**Table 2:** Clinical trials of epi-drugs in breast cancer.

Phase(NCT No./Ref.)	Patient Population (*n*)	Interventions	Outcome	Status	Date Started	Estimated Completion Date
**DNMT Inhibitor**						
Phase I ([53])	Breast cancer/Solid tumor (4/19)	Decitabine, continuous infusion	No responses in breast cancer	Completed		
Phase I ([54])	Breast cancer/Solid tumor (5/33)	Decitabine + Carboplatin	No responses in breast cancer	Completed		
Phase I (NCT00748553/[55])	Breast cancer/Solid tumor (1/16)	Azacitidine + nab-paclitaxel	1 PR in Breast cancer	Completed		
Phase II (NCT01349959)	Hormone-resistant breast cancer (27) and TNBC (13)	Azacitidine + Entinostat	1 PR (1/27, 4%) in hormone-resistant breast cancer No responses in TNBC	Completed		
Phase II (NCT02811497)	Unspecified solid tumor including ER(+) HER2(−) breast cancer	Azacitidine + Durvalumab	Overall response rate	Active, not recruiting	September 2016	January 2022
Phase II (NCT02957968)	HER2(−) breast cancer	Decitabine + Neoadjuvant Pembrolizumab	Changes of tumor infiltrating lymphocytes	Recruiting	January 2017	February 2023
**HDAC Inhibitor**						
Phase II (NCT00404508)	Breast cancer/Solid tumor (3/15)	Valproate + Hydralazine + chemotherapy	1 SD (1/3, 33%) in breast cancer	Completed		
Phase I ([56])	Breast cancer/Solid tumor (10/44)	Valproate + Epirubicin	3 SD (3/10, 30%) and 3 PR (3/10, 30%) in breast cancer	Completed		
Phase I (NCT00878904)	Breast cancer/Solid tumor (5/37)	Panobinostat + Epirubicin	2 PR (2/5, 40%) in breast cancer	Completed		
Phase II (NCT00676663/[57])	ER(+) breast cancer (130)	Entinostat + Exemestane	PFS: 4.3 vs. 2.3 months OS: extend 28.1 vs. 19.8 months	Completed		
Phase III (NCT02482753/[58])	ER(+) breast cancer (365)	Tucidinostat + Exemestane	PFS: 7.4 vs. 3.8 months Common hematological adverse events	Completed		
Phase II (NCT00258349/[59])	HER2(+) breast cancer (16)	Vorinostat + Trastuzumab	No response	Completed		
Phase II (NCT00365599/[60])	HR(+) breast cancer (43)	Vorinostat + Tamoxifen	Objective response: 8/43, 19%	Completed		
Phase II (NCT02395627/[61])	ER(+) breast cancer (34)	Vorinostat + Tamoxifen + Pembrolizumab	Objective response: 1/27 (4%) Clinical benefits: 5/27 (18%)	Terminated		
Phase II (NCT02453620)	HER2(−) breast cancer	Entinostat + Nivolumab + Ipilimumab	AE	Active, not recruiting	November 2015	December 2020
Phase II (NCT03280563)	HR(+) HER2(−) breast cancer	Entinostat + Atezolizumab	Objective response	Recruiting	December 2017	October 2022
Phase II (NCT04190056)	ER(+) breast cancer	Vorinostat + Tamoxifen + pembrolizumab	Overall response rate	Not yet recruiting	December 2020	June 2026
Phase I (NCT04296942)	TNBC or HR(−) HER2(+) breast cancer	Entinostat + BN-Brachyury + Adotrastuzumab emtansine + M7824	Overall response rate	Not yet recruiting	November 2020	January 2022
**LSD1 Inhibitor**						
Phase I (NCT03505528)	HER2(−) breast cancer	Phenelzine + Nab-paclitaxel	Dose-limiting toxicity (not reported yet)	Completed		
**BET Inhibitor**						
Phase I/II (NCT01587703/[62])	TNBC/*NUT* carcinoma or Solid tumor (5/65)	Molibresib	1 SD (1/5, 20%) and 1 PR (1/5, 20%) in breast cancer Thrombocytopenia (51%)	Completed		
Phase I (NCT02391480/[63])	TNBC/Solid tumor (8/72)	Mivebresib	Grade 3 or 4 AE: 57% 1 SD (1/8, 16%) in breast cancer	Completed		
Phase II (NCT03901469)	TNBC	ZEN003694 +Talazoparib	Dose-limiting toxicities Objective response	Recruiting	June 2019	January 2022
Phase I/II (NCT02419417)	Unspecified advanced cancer	BMS-986158 + Nivolumab	AE	Recruiting	June 2015	July 2023

Abbreviations: AE: adverse events; ER: estrogen receptor; PR: progesterone receptor; HR: hormone receptor; HER2: human epidermal growth factor receptor 2; PFS: progression-free survival; PR: partial response; SD: stable disease; TNBC, triple-negative breast cancer.
